# Obituary

**Published:** 1886-12

**Authors:** 


### Editorial.

### Obituary.

Died, on Wednesday, Nov. 24, Cincinnati, Ohio, Dr. Edmund Osmond. The Dr. had been for a number of months in declining health. He had been subject to asthma for many years, this, together with some other difficulties, resulted in his death, as has been stated. Dr. Osmond was a native of Hereford, England; was born October 8, 1828, and came to the United States in 1850. He practiced medicine and surgery in Sidney, Ohio, about five years. He then came to Cincinnati, entered the Ohio College of Dental Surgery, from which he graduated, March, 1856, immediately after which he located in Cincinnati, where he has practiced dentistry continuously until the time of his death.

Dr. Osmond married Miss E. A. Edwin, of England, September 21st, 1867, who, with three daughters, survive to mourn his loss.

Dr. Osmond was widely known in the profession as a thorough, enterprising practitioner of dentistry. He devised a number of instruments and processes of much value. The improved hand piece for the dental engine was one of these. He also devised a set of instruments for using gold screws, to be used in large and difficult fillings of cavities in decayed teeth.

He was well up in electrical science, which was fully shown in the applications which he made of this agent to various parts of dental practice. He invented and constructed an electric battery, superior to anything hitherto in use. He was fertile in devices and resources.

The following action had by the dentists of Cincinnati, at a recent meeting, fully shows the esteem in which he was held by his profession:

At a meeting of the dentists of the city, held yesterday afternoon, at the office of Dr. A. Berry, that gentleman presiding, and Dr. H. L. Moore acting as Secretary, there being present besides, Drs. J. Taft, H. A. Smith, C. M. Wright, Frank A. Hunter, O. N. Heise, Charles Junkerman, R. J. Poore, H. A. Downing, R. E. Taylor, M. H. Fletcher, Frank Sage, Wm. Taft, E. G. Betty: the following setting forth the feeling in regard to the death of Dr. Osmond, was adopted:

"Whereas, We have received the sad intelligence of the death of Dr. E. Osmond, who has long been identified with the interests of the dental profession in this city; and

"Whereas, We, as members of the dental profession, here assembled, deeming it most fitting and proper to give an expression of the esteem and regard in which the deceased was held, adopt the following:

"*Resolved*, That we most fully realize that in the death of Dr. Osmond the profession has lost a faithful member, one who was justly entitled to the respect and regard of all who knew him. He always evinced a great interest in all that pertained to his chosen profession, as a result of which, in several particulars, much was by his skill and genius added to its resources. Its progress was ever a matter of solicitude to him. For his friends he had an unusually strong attachment, and in every way he was a a person of strong and decided views.

"*Resolved*, That we tender to the family our most sincere and heartfelt sympathy in this their great bereavement and sorrow.

"*Resolved*, That a copy of these resolutions, be sent in proper form to the family, and copy furnished to the press for publication."

